# Successful Repair of Infantile Scimitar Syndrome with Contralateral Pulmonary Vein Stenosis in an Infant

**DOI:** 10.21470/1678-9741-2019-0228

**Published:** 2021

**Authors:** Mehmet Akif Onalan, Bahar Temur, Selim Aydın, Ersin Erek

**Affiliations:** 1 Department of Cardiovascular Surgery, Faculty of Medicine, Acibadem Mehmet Ali Aydinlar University, Istanbul, Turkey.

**Keywords:** Pulmonary Syndrome, Pulmonary Veins, Respiratory System Abnormalities, Heart Defects, Congenital, Survivors

## Abstract

Infantile scimitar syndrome (SS) is a rare congenital heart disease and has high mortality. Guidelines have not been established, but surgery is indicated in symptomatic patients. Despite the various surgical approaches, outcomes continue to be disappointing. We present our surgical experience with an infantile SS patient who had stenotic pulmonary veins contralateral to the hypoplastic lung with complicated anatomy. There are few cases with this complex pathology in the literature. Moreover, our patient was the first transplant-free survivor with this complexity in the literature.

**Table:** 

Abbreviations, acronyms & symbols	
ASD	= Atrial septal defect
CPB	= Cardiopulmonary bypass
ECHO	= Echocardiogram
ECHSA	= European Congenital Heart Surgeons Association
ICU	= Intensive care unit
LA	= Left atrium
LLPV	= Left lower pulmonary vein
LPV	= Left pulmonary vein
PDA	= Patent ductus arteriosus
PHT	= Pulmonary hypertension
PVS	= Pulmonary vein stenosis
RSPV	= Right superior pulmonary vein
SS	= Scimitar syndrome
TAPVC	= Total anomalous pulmonary venous connection
VSD	= Ventricular septal defect

## INTRODUCTION

Scimitar syndrome (SS) is an unusual condition of anomalous pulmonary venous return from the right lung to the inferior vena cava. The infantile form presents early in life with symptoms of tachypnea, chest infection, heart failure, and failure to thrive, and, as a result, it always requires intervention^[1]^.

Primary (native or congenital) pulmonary vein stenosis (PVS) is a rare, challenging, and usually frustrating disease^[2]^. Although there are some reports showing stenosis of the scimitar vein, the involvement of contralateral PVS is very rare. To the best of our knowledge, only three other patients have been described with stenosis of the pulmonary veins contralateral to the hypoplastic lung^[1,3,4]^. We report the successful surgical management of an infant with infantile SS and contralateral PVS.

## CASE REPORT

**Fig. 1:**
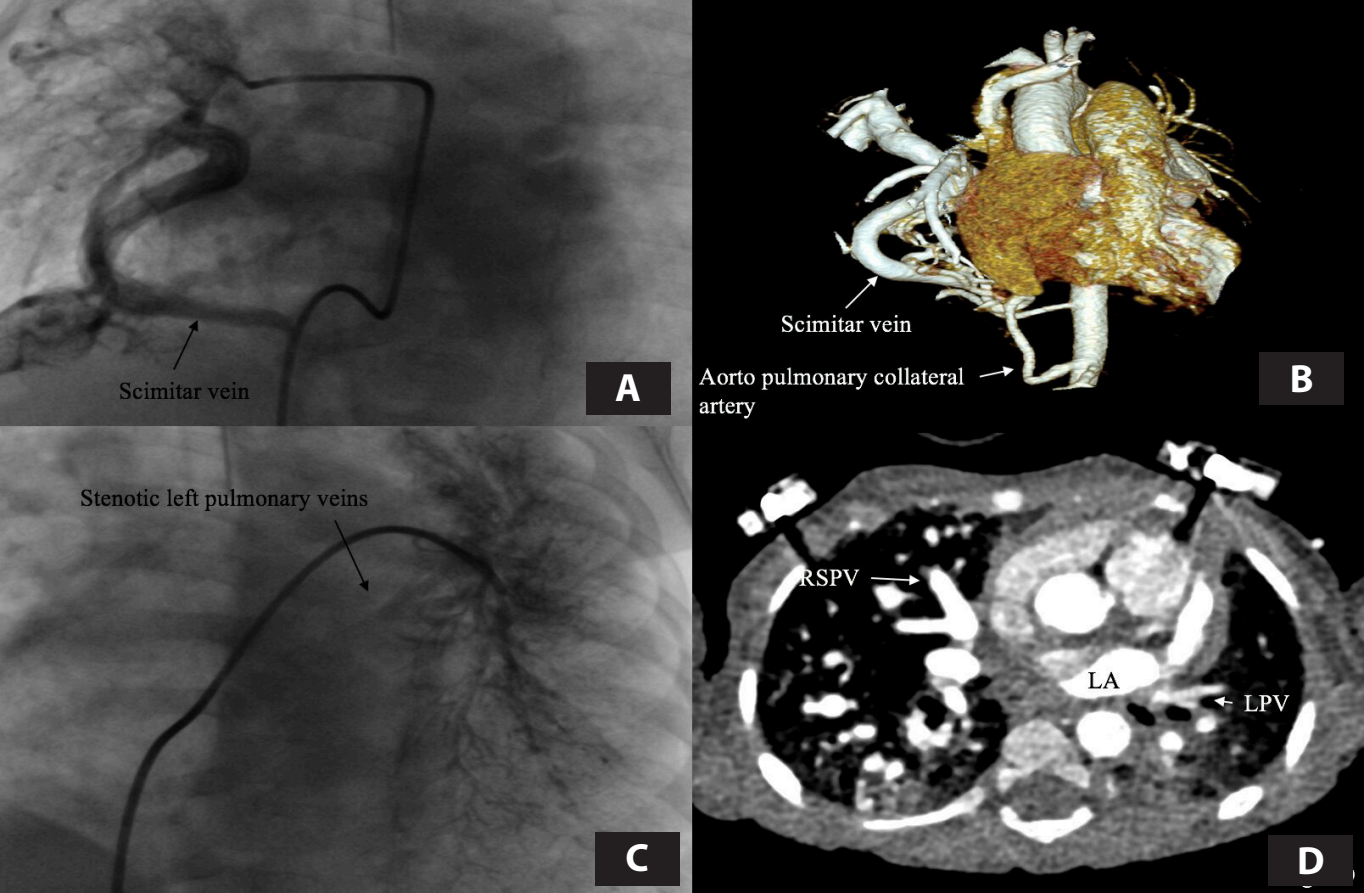
Preoperative computed tomography angiography and cardiac catheterization demonstrating the right lower anomalous pulmonary venous return to the inferior vena cava via the scimitar vein, the aortopulmonary collateral artery from the abdominal aorta to the right lower lung (A,B), and the stenotic left pulmonary veins (C,D). LA=left atrium; LPV=left pulmonary vein; RSPV=right superior pulmonary vein

A female patient, aged 13 months, was referred to our clinic with a diagnosis of SS, muscular ventricular septal defect (VSD), and pulmonary hypertension (PHT). She was suffering from respiratory distress, was tachypneic, and required continuous nasal oxygen support. She had been operated because of right diaphragmatic hernia at three months of age. Her body weight was 4800 g. The chest radiograph showed an irregular shadow in the right lower lung and cardiomegaly. A transthoracic M-mode, two-dimensional, color-flow Doppler echocardiogram (ECHO) showed right lower anomalous pulmonary venous return entering a dilated inferior vena cava via the vertical vein (scimitar vein) with systemic PHT (mean 70 mmHg), a moderately hypoplastic right lung, muscular VSD (5 mm in size), and a moderately sized patent ductus arteriosus (PDA). Turbulent flow was detected where the left pulmonary veins poured into the left atrium. Substantial dilation of the right atrium and the right ventricle were also present. Computed tomography angiography and cardiac catheterization confirmed the echocardiographic findings (Figure 1). The patient’s Aristotle Comprehensive Complexity score was 8.0. Ostial stenosis of the left pulmonary veins was detected, and the aortopulmonary collateral artery from the abdominal aorta to the right lower lung was coil embolized during catheterization.

**Fig. 2:**
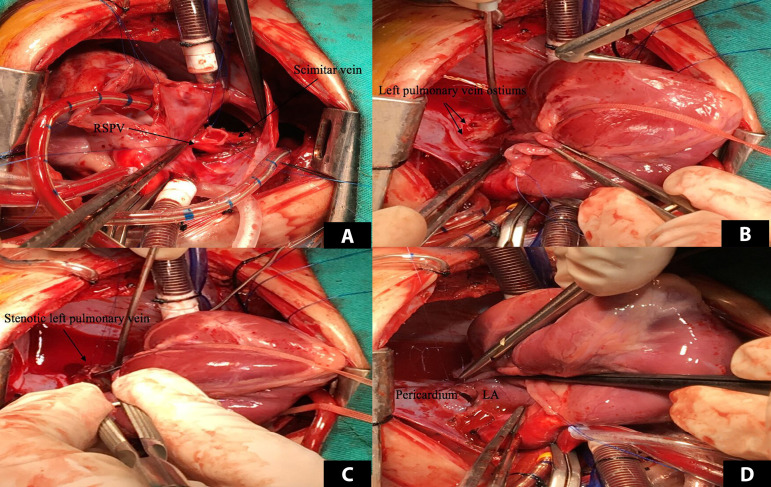
A) End-to-side anastomosis of the scimitar vein to the right upper pulmonary vein. B and C) Ostial stenosis of the left pulmonary veins. D) Suturing the upper part of the left atrial incision to the pericardium and covering the left pulmonary veins without sutures. LA=left atrium; RSPV=right superior pulmonary vein

Surgical repair was undertaken. The PDA was ligated, and the muscular VSD was closed. The scimitar vein was dissected and ligated at the diaphragm level. It was end-to-side anastomosed to the right upper pulmonary vein. The left pulmonary vein orifices were observed from the left atrium via an atrial septal defect (ASD). Pinpoint stenosis was detected. The heart was tilted to the right. The left pulmonary veins were incised at the hilum. Incisions were advanced into the left atrium. The left atrium was opened vertically just above the left pulmonary vein entries. The upper part of the left atrial incision was sutured generously to cover the left pulmonary veins without suturing the pulmonary veins as a sutureless repair^[5]^ (Figure 2). A 4-mm patent foramen ovale was left open. Cessation of cardiopulmonary bypass was uneventful, and the sternotomy was closed. The patient was transferred to the cardiovascular intensive care unit (ICU) with moderate doses of inotropic support and in stable hemodynamic condition. Nitric oxide inhalation, iloprost infusion, and sildenafil were started in the ICU to control PHT. Control ECHO examination revealed no stenosis of the left or right pulmonary veins. Several attempts failed to wean the patient from respiratory support. Cardiac catheterization was performed to control pulmonary venous circulation on day 15, and no stenosis was detected, but persistent mild PHT was present. The patient received antibiotics due to pneumonia, and a tracheostomy was performed. She was transferred to a ward with a home ventilator system. She was gradually weaned from ventilatory support and eventually discharged from the hospital on postoperative day 95 with the tracheostomy in place. The patient’s characteristics and outcomes are listed in Table 1 in chronological order. The patient’s Technical Performance Score was calculated as class 1 based on echocardiographic and clinical findings at discharge. Despite occasional respiratory distress, she did not require hospitalization during the six-month follow-up period.

**Table 1:** Patient's characteristics and outcomes.

Age at surgery, months	13
Body weight at surgery, kg	4.8
CPB time, min	110
Circulatory arrest time, min	23
ICU stay, days	68
Hospitalization, days	95
Follow-up, months	6

CPB=cardiopulmonary bypass; ICU=intensive care unit

## DISCUSSION

Infantile SS is very rare, and no single center has brought together a large series of patients. Therefore, the chance to study this syndrome is limited, and the literature on infantile SS consists of case reports and small case series^[1]^. Currently, there is no consensus on the best treatment option. Nonetheless, the existence of PHT and non-response to medical therapy are considered indications for intervention^[2]^. A two-stage approach is recommended in infants with coiling of the arterial collaterals and surgically repairing the anomalous vein^[1]^.

The association between infantile-type SS and contralateral PVS is very rarely reported and has dismal results. In this case, scimitar vein reimplantation to the left atrium and sutureless repair for left pulmonary veins were performed successfully.

To the best of our knowledge, only three cases of infantile SS with PVS contralateral to the hypoplastic lung have been described. Gao et al.^[3]^ reported a four-month-old patient who had severe PHT. He was treated with surgical ligation of the systemic feeding artery and balloon dilatation of the left common PVS. However, the severe PHT did not resolve and the patient died a few days after unsuccessful balloon dilatation at two years of age. Huddleston et al.^[4]^ reported a four-month-old patient with bilateral PVS associated with SS. They performed complete repair through atrial baffling of the anomalous pulmonary vein and intraoperative pulmonary vein stent placement. The patient, who underwent successful bilateral lung transplant, was in good clinical condition two years after the transplant^[4]^. The last three-month-old patient was reported by Argueta-Morales et al.^[1]^ They performed embolization of the aortopulmonary collateral artery, and surgical repair was undertaken at 6.5 months old, with ASD, VSD, and PDA closure and right ventricle outflow tract muscle resection. Eight days after discharge, the patient needed rehospitalization because of PHT and decreased right ventricle function. Left lower pulmonary vein (LLPV) ostial stenosis was detected by cardiac catheterization, and stent implantation was performed into the proximal LLPV. Because of unsatisfactory results, a second surgical intervention was performed with stent removal and left-side sutureless pericardial repair. The patient died of sepsis at 10 months of age.

Pulmonary arterial hypertension with infantile-type SS has severe symptoms and poor outcomes^[6]^. The European Congenital Heart Surgeons Association (ECHSA) multicentric study data, which consisted of 68 patients who underwent surgery for SS between January 1997 and December 2007 from 19 cardiothoracic centers, reported that the presence of pulmonary arterial hypertension is associated with higher mortality rate^[7]^. Hospital mortality and late deaths were significantly higher in patients with pulmonary arterial hypertension in that study^[7]^. These data agreed with the higher postoperative mortality and morbidity rates, confirming previous studies^[6]^. In our case, persistent mild PHT caused a prolonged stay in the ICU and hospital.

Management of PVS is very challenging, and most of the experience comes from the total anomalous pulmonary venous connection (TAPVC) series. The optimal results from “sutureless” repair for the relief of postoperative PVS in patients undergoing surgical repair of TAPVC have been reported frequently^[6]^. The sutureless technique with pericardial marsupialization was introduced as a method to relieve stenosis while minimizing injury to the pulmonary vein wall by avoiding direct suturing of the vein^[5]^. The other traditional repair is conventional relief of PVS, such as cutback, resection of stenotic intima, or relocation^[6]^. Yamashita et al.^[8]^ conducted a study with 12 patients who underwent TAPVC repair from 1999 to 2011 and compared outcomes between conventional procedures and sutureless techniques. This study reported the superiority of the sutureless technique in terms of the relief of the stenosed pulmonary vein^[8]^. Stent implantation for the relief of PVS, either surgical or by catheterization, usually has disappointing results. In our case, we preferred the reimplantation technique to repair the scimitar vein and performed a sutureless technique with left-sided marsupialization^[5]^ to relieve contralateral PVS. Postoperatively, no stenosis was detected in pulmonary veins by ECHO or control catheterization.

Infantile SS is a very rare congenital anomaly that needs to be diagnosed early and treated in time. Contralateral PVS should be kept in mind during diagnostic evaluation. We reported the successful surgical management of infantile SS with contralateral PVS. Although the follow-up period is short, our patient is the first transplant-free survivor in the literature.

**Table:** 

Author's roles & responsibilities	
MAO	Substantial contributions to the conception or design of the work; or the acquisition, analysis, or interpretation of data for the work; drafting the work or revising it critically for important intellectual content; final approval of the version to be published
BT	Drafting the work or revising it critically for important intellectual content; agreement to be accountable for all aspects of the work in ensuring that questions related to the accuracy or integrity of any part of the work are appropriately investigated and resolved; final approval of the version to be published
SA	Substantial contributions to the conception or design of the work; or the acquisition, analysis, or interpretation of data for the work; agreement to be accountable for all aspects of the work in ensuring that questions related to the accuracy or integrity of any part of the work are appropriately investigated and resolved; final approval of the version to be published
EE	Substantial contributions to the conception or design of the work; or the acquisition, analysis, or interpretation of data for the work; drafting the work or revising it critically for important intellectual content; final approval of the version to be published
